# Supplementary material for: Causal Relationship between Aging and Anorexia Nervosa: A White-Matter-Microstructure-Mediated Mendelian Randomization Analysis
Source: Biomedicines. 2024 Aug 16;12(8):1874. doi: 10.3390/biomedicines12081874 (PMC11351342; doi:10.3390/biomedicines12081874)
Supplement: Supplementary file 1 [file biomedicines-12-01874-s001.zip › Supplementary Figure/Figure S8/Leaveoneout plot of MR analysis for TL to ubm-b-1953.pdf]

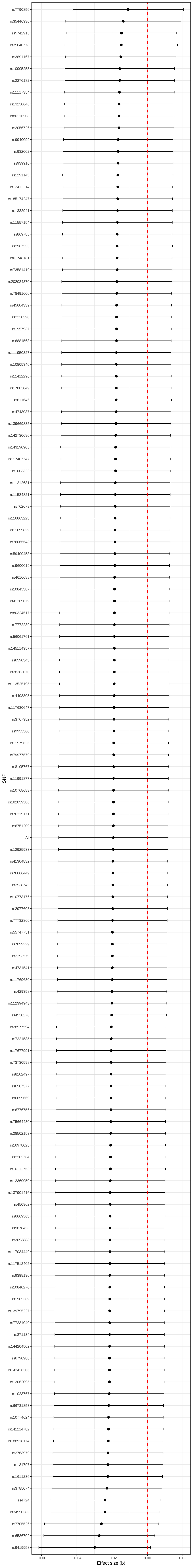

rs7790856
rs35446936
rs5742915
rs35640778
rs3891167
rs10905255
rs2276182
rs11117354
rs13230646
rs80116508
rs2056726
rs9940099
rs932002
rs939916
rs1291143
rs12412214
rs185174247
rs1332941
rs11557154
rs869785
rs2967355
rs67748181
rs73581419
rs202034370
rs78491606
rs45604339
rs2230590
rs1957937
rs6881568
rs111950327
rs10805346
rs11412296
rs17803849
rs611646
rs4743037
rs139669835
rs142730696
rs143190905
rs117407747
rs1003322
rs11212631
rs11584821
rs762679
rs116863223
rs11699829
rs76065543
rs59409453
rs9600019
rs4616688
rs10845387
rs41269079
rs80324517
rs7772289
rs56061761
rs145114957
rs6590343
rs28363070
rs113525195
rs4498805
rs117630647
rs3767952
rs9955360
rs11579626
rs79977579
rs8105767
rs11991877
rs10768683
rs182059586
rs76219171
rs6751209
All
rs12925933
rs41304832
rs76666449
rs2538745
rs10773176
rs2977608
rs77732866
rs55747751
rs7099229
rs2293579
rs4731541
rs11769630
rs429358
rs112394943
rs4530278
rs28577594
rs7221585
rs17677991
rs73730598
rs8102497
rs6587577
rs6659669
rs6776756
rs75664430
rs28502153
rs16978028
rs2282764
rs10112752
rs12369950
rs137901416
rs450962
rs6669563
rs9878436
rs3093888
rs117034449
rs117512405
rs9398196
rs10840270
rs1985369
rs139795227
rs77231040
rs871134
rs144204502
rs6790988
rs142426306
rs13062095
rs1023767
rs66731853
rs10774624
rs141214782
rs188918174
rs2763979
rs131797
rs11611236
rs3785074
rs4724
rs34550383
rs7705526
rs6536702
rs9419958
SNP
-0.06
-0.04
-0.02
0.00
0.02
Effect size (b)
